# Non-competitive NMDA antagonist MK-801 prevents memory reconsolidation impairment caused by protein synthesis inhibitors in young chicks

**DOI:** 10.3389/fphar.2024.1378612

**Published:** 2024-07-04

**Authors:** A. A. Tiunova, D. V. Bezriadnov, K. V. Anokhin

**Affiliations:** Institute for Advanced Brain Studies, Lomonosov Moscow State University, Moscow, Russia

**Keywords:** memory, reconsolidation, chicks, NMDA antagonists, protein synthesis

## Abstract

**Introduction:** Reactivation of already consolidated memory can initiate its destabilization, making the memory trace labile. Normally, this destabilization is followed by reconsolidation of the memory trace, enriched by newly acquired experience. Disrupting the reconsolidation process, for example, by inhibiting protein synthesis, impairs subsequent memory retrieval, leading to reminder-related amnesia. Previous studies in various species have shown that this impairment can be prevented by using NMDA receptor antagonists, which interfere with memory destabilization.

**Methods:** In the present study we examined this phenomenon using a one-trial passive avoidance learning model in newborn chicks, the hypothesis being that inactivation of the NMDA-mediated transmission during memory reactivation would inhibit the memory trace labilization and thus prevent the reminder-related amnesia.

**Results:** We found that reminder-associated administration of the NMDA receptor antagonist MK-801 or one of the protein synthesis inhibitors (anisomycin, cycloheximide, 2-deoxygalactose) each alone produced amnesia. However, when combined, injection of MK-801 before the reminder prevented amnesia induced by protein synthesis inhibitors.

**Discussion:** We suggest that the observed paradoxical effect implicates the involvement of NMDA receptors in both protein synthesis-independent engram destabilization upon its retrieval and protein synthesismediated engram stabilization after its updating. This puzzling dual role of NMDA receptors in memory destabilization/restabilization requires further investigation.

## Introduction

Memory reactivation is known to induce destabilization of the memory trace, followed by reconsolidation of the updated memory (Bellfy and Kwapis, 2020). Interference with the reconsolidation process can impair the memory trace. Various studies have shown that reminder-associated amnesia can be induced across multiple species using protein synthesis inhibitors, glycosylation inhibitors, glutamate receptor antagonists, PKA inhibitors, CREB suppression, and MAPK inhibitors (Przybyslawski and Sara, 1997; Anokhin et al., 2002; Kida et al., 2002; Kelly et al., 2003; Koh and Bernstein, 2003; Ben Mamou et al., 2006). This amnesia can be transient or permanent, recoverable or irreversible, and is influenced by factors such as the pharmacological agent, timing, experimental paradigm, and species. It has been interpreted as disruption of memory trace, prevention of access, or retrieval failure (Sara and Hars, 2006; Crowe et al., 2008; Haubrich et al., 2020). Some studies have demonstrated that reminder-associated amnesia can be prevented by administering another amnestic agent before the reminder. In particular, a number of studies have examined the role of NMDA receptors in the processes of memory destabilization (labilization) and restabilization (reconsolidation). NMDA receptors, which belong to the ionotropic glutamate receptors, have been shown to play a crucial role in synaptic plasticity (Paoletti et al., 2013; Sanz-Clemente et al., 2013). For example, amnesia induced by protein synthesis inhibitors in rats was prevented by NMDA receptor antagonists (Ben Mamou et al., 2006), and similar results were observed with different receptor antagonists in other species (Solntseva and Nikitin, 2011; Nikitin and Solntzeva, 2012; Balaban et al., 2014; Bal et al., 2017; Rossato et al., 2023). These findings suggest that inhibiting memory destabilization during retrieval can prevent its subsequent impairment. Recent studies have also explored the distinct roles of NMDA receptor subunits in memory destabilization and reconsolidation (Milton et al., 2013; Wideman et al., 2020; Radiske et al., 2021; Rossato et al., 2023). In our current study, based on the hypothesis that inactivation of the NMDA receptors during memory reactivation would inhibit the memory trace labilization, we investigated the potential to prevent reminder-associated amnesia in avian species, using a one-trial learning model in newborn chicks.

## Materials and methods

The study was carried out in accordance with the recommendations of the Directive 2010/63/EU of the European Parliament and of the Council of the European Union issued 22 September 2010, on the protection of animals, used for scientific purposes (Section 27). The protocol was approved by the Animal Ethics committee of the Lomonosov Moscow State University.

Day-old domestic chicks of both sexes (Panzirevskaya Black strain) were obtained from the Research and Technological Poultry Institute, Moscow Region. They were housed in pairs in metal pens (20 × 25 × 20 cm) and acclimatized overnight with access to food and water. The experimental room was maintained at 30°C with a 12:12 h dark/light cycle. The experiments commenced the following morning, when the chicks reached the age of 2 days and their weight ranged from 36 to 40 g. Chicks were pre-trained with two 10-s presentations of a 2-mm dry metal bead on a rod. Only chicks that pecked at the bead (over 90% of them) proceeded to the next stage. Twenty minutes after the pre-training, the chicks underwent training with a 2-mm white plastic bead on a rod coated with methyl anthranilate (MeA; Sigma), a bitter substance. Chicks that pecked the bead exhibited a disgust reaction (head shaking and beak wiping) and subsequently avoided pecking an identical but dry bead during the retention test. Training of chicks in a passive avoidance model leads to memorizing and subsequent avoidance of the specific bead that was used for training (Crowe et al., 2008; Krolik et al., 2020; Tiunova et al., 2020; for a review, see Rose, 2000; Matsushima et al., 2003). In the standard paradigm we used, a neutral bead of a different color was presented 20 min after memory test for the aversive bead, and only those chicks that pecked the neural bead and avoided the aversive bead were included in the analysis.

Two hours post-training, the chicks received a reminder, which involved presenting a dry white bead identical to the training bead. If a chick did not peck within 10 s, this was classified as “avoidance” since typically it was accompanied by manifestations of a noticeable avoidance behavior, such as backing away and distress calls. The response of pecking or avoiding the dry bead was recorded. Over 80% of the chicks displayed avoidance, exhibiting disgust behavior; those that pecked were excluded from further experiments. Two hours after the reminder (i.e., 4 h post-training), the chicks were tested for retention. The testing procedure was identical to the reminder, involving presentation of the same dry white bead. Responses (peck or avoid) were recorded, and a percentage avoidance score was calculated for each experimental group as a proportion of avoiding animals (e.g., Krolik et al., 2020). The avoidance levels between groups were compared using the χ2 test of independence. Differences were considered significant at *p* < .05. To ensure that the chicks did not develop a generalized avoidance reaction, they were also presented with the dry chrome bead used in pre-training; only chicks that pecked at this bead were included in the analysis. Each chick was used only once for training, reminder, and testing. All behavioral procedures were carried out by a researcher blind to the injected solutions, and the person conducting the test did not know which experimental group each chick belonged to.

In total, 510 chicks were taken for the experiments and 477 of them were used in the data analysis. The withdrawal (<6.5%) was applied to the chicks that did not peck in the pre-training or training trials and to those which pecked at the aversive bead during the reminder session.

All chicks underwent pre-training and training for passive avoidance. Two hours post-training, the chicks in the experimental groups received a reminder. In these groups, each chick was administered MK-801 or saline intraperitoneally 30 min before the reminder, followed by an intraventricular injection of anisomycin (ANI), cycloheximide (CXM), 2-deoxygalactose (2-D-Gal), or saline 5 min prior to the reminder. Control chicks received neither reminders nor drugs and were tested alongside the experimental groups 4 h after training.

Anisomycin (80 μg, Sigma), cycloheximide (20 μg, Serva), and 2-deoxygalactose (3.28 mg, Sigma) were administered intraventricularly either 5 min before or immediately after the reminder. Bilateral intracranial injections (5 μL per hemisphere) were performed using a 10-μL syringe and a specialized headholder to target the lateral ventricles and adjacent brain areas (Davis et al., 1979). MK-801 ((+)-MK-801 hydrogen maleate, Sigma) was administered intraperitoneally at a dose of 0.25 mg/kg in 0.1 mL saline, 30 min before the reminder. Post-experiment, injection sites were routinely inspected.

## Results

**FIGURE 1:**
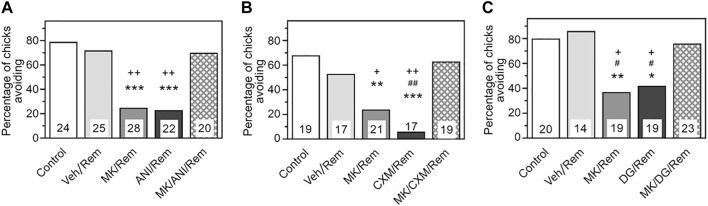
Administration of NMDA antagonist MK-801 prior to reminder prevents amnesia produced by pre-reminder administration of a protein synthesis inhibitor. Data are shown as the percentage of chicks showing avoidance. Numbers of chicks in each group are shown in the bars. **(A)** Anisomycin (ANI). Experimental groups: Control–Training and retention test; Veh/Rem–Training, Reminder 2 h post-training coupled with vehicle injection, retention test; MK/Rem–Training, Reminder 2 h post-training coupled with MK-801 injection, retention test; ANI/Rem—Training, Reminder 2 h post-training coupled with anisomycin injection, retention test; MK/ANI/Rem—Training, Reminder 2 h post-training coupled with MK-801 and anisomycin injections, retention test. Avoidance levels in the retention test 4 h after the training. MK-801 administered 30 min pre-reminder, ANI—5 min pre-reminder. ***p < 0.001 compared to Control and Veh/Rem groups; ^++^ *p* < 0.01compared to MK/ANI/Rem group. **(B)** Cycloheximide (CXM). Experimental groups: Control–Training and retention test; Veh/Rem–Training, Reminder 2 h post-training coupled with vehicle injection, retention test; MK/Rem–Training, Reminder 2 h post-training coupled with MK-801 injection, retention test; CXM/Rem—Training, Reminder 2 h post-training coupled with cycloheximide injection, retention test; MK/CXM/Rem—Training, Reminder 2 h post-training coupled with MK-801 and cycloheximide injections, retention test. Avoidance levels in the retention test 4 h after the training. MK-801 administered 30 min pre-reminder, CXM– 5 min pre-reminder. **p < 0.01, ***p < 0.001 compared to Control group; ^##^ *p* < 0.01 compared to Veh/Rem group; ^+^ *p* < 0.05, ^++^ *p* < 0.01 compared to MK/CXM/Rem group. **(C)** 2-deoxygalactose (DG). Experimental groups: Control–Training and retention test; Veh/Rem–Training, Reminder 2 h post-training coupled with vehicle injection, retention test; MK/Rem–Training, Reminder 2 h post-training coupled with MK-801 injection, retention test; DG/Rem—Training, Reminder 2 h post-training coupled with 2-deoxygalactose injection, retention test; MK/DG/Rem—Training, Reminder 2 h post-training coupled with MK-801 and 2-deoxygalactose injections, retention test. Avoidance levels in the retention test 4 h after the training. MK-801 administered 30 min pre-reminder, DG – 5 min pre-reminder. *p < 0.05, **p < 0.01 compared to Control group; ^#^ *p* < 0.05 compared to Veh/Rem group; ^+^ *p* < 0.05, compared to MK/DG/Rem group.

As illustrated in Figure 1A, the avoidance level during the recall test was high in chicks receiving a reminder combined with saline injections (Reminder group, N = 25), showing no significant difference from the group receiving no reminder (Control, N = 24). Administration of MK-801 prior to the reminder led to a marked retention deficit (MK/Reminder group, N = 28, *p* < .001 compared to the Control), corroborating previous findings in chicks (Summers et al., 1995; Summers et al., 1997) and rodents (Przybyslawski and Sara, 1997). Similarly, pre-reminder administration of anisomycin significantly decreased avoidance levels (ANI/Reminder group, N = 22, *p* < .001 compared to the Control), confirming earlier studies on reminder-associated amnesia (Anokhin et al., 2002). However, in the group receiving both MK-801 and ANI, retention remained unimpaired, matching the control group (MK/ANI/Reminder group, N = 20, *p* < .001 compared to MK/Rem and ANI/Rem groups).

Considering potential side effects of anisomycin beyond protein synthesis inhibition (Remaud et al., 2014), we investigated whether MK-801 could prevent the amnestic effects of a different protein synthesis inhibitor (PSI), cycloheximide (Figure 1B). Administration of either MK-801 or cycloheximide alone prior to the reminder impaired performance in the retention test 2 h post-reminder (MK/Reminder, N = 21, *p* < .01 compared to the Control, and CXM/Reminder groups, N = 17, *p* < .001 compared to the Control). However, administration of both MK-801 and cycloheximide abolished the amnestic effect (MK/CXM/Reminder group, N = 19, *p* < .05 compared to the MK/Rem and *p* < .01 compared to the CXM/Rem).

Thus, the NMDA antagonist MK-801 prevented reminder-associated amnesia caused by two different PSIs. We further assessed the impact of MK-801 on amnesia induced by 2-deoxygalactose, an inhibitor of post-translational protein fucosylation (Anokhin et al., 2002). Administration of 2-D-Gal 5 min before the reminder significantly reduced avoidance levels tested 2 h post-reminder (Figure 1C, DG/Reminder group, N = 19, *p* < .05 compared to the Control). Pre-reminder injection of MK-801 impeded this effect (MK/DG/Reminder group, N = 23, *p* < .05 compared to DG/Rem group), so that this group did not differ from the Control and Reminder groups (N = 20, N = 14 respectively).

**FIGURE 2:**
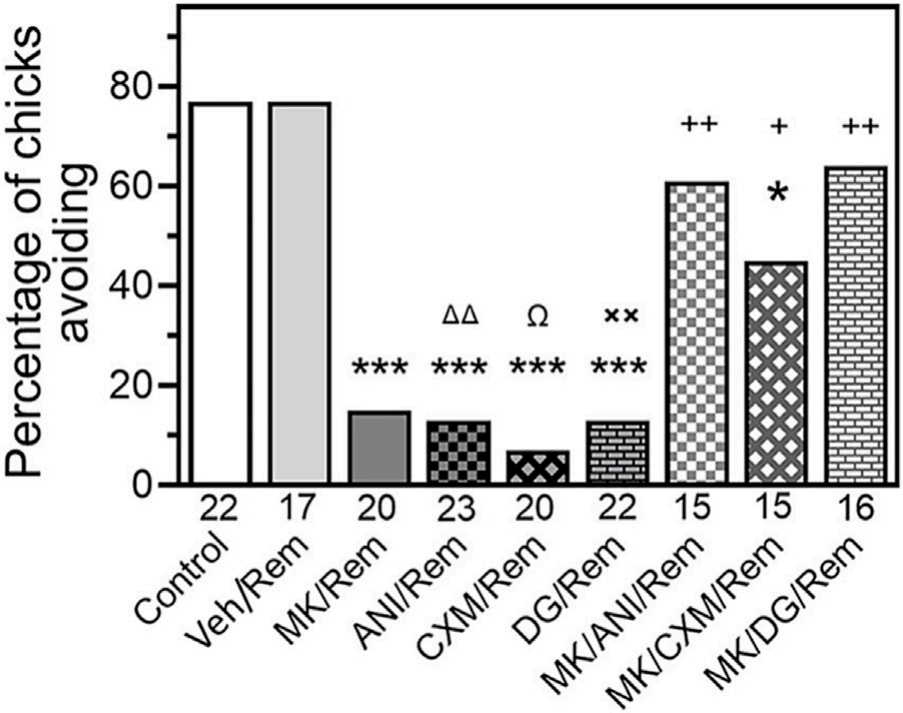
Administration of NMDA antagonist MK-801 prior to reminder prevents amnesia produced by post-reminder administration of a protein synthesis inhibitor. Data are shown as the percentage of chicks showing avoidance. Numbers of chicks in each group are shown under the bars. Experimental groups: Control–Training and retention test; Veh/Rem–Training, Reminder 2 h post-training coupled with vehicle injection, retention test; MK/Rem–Training, Reminder 2 h post-training coupled with MK-801 injection, retention test; ANI/Rem - Training, Reminder 2 h post-training coupled with post-reminder anisomicyn injection, retention test; DG/Rem—Training, Reminder 2 h post-training coupled with post-reminder 2-deoxygalactose injection, retention test; CXM/Rem—Training, Reminder 2 h post-training coupled with post-reminder cycloheximide injection, retention test; MK/ANI/Rem—Training, Reminder 2 h post-training coupled with MK-801 and anisomicyn injections, retention test. MK/DG/Rem—Training, Reminder 2 h post-training coupled with MK-801 and 2-deoxygalactose injections, retention test. MK/CXM/Rem—Training, Reminder 2 h post-training coupled with MK-801 and cycloheximide injections, retention test. Avoidance levels in the retention test 4 h after the training.MK-801 administered 30 min pre-reminder, ANI, DG and CXM – 5 min after the reminder. *p < 0.05 compared to Control group; ***p < 0.001 compared to Control and Veh/Rem groups; ^ΔΔ^P<0.01 compared to MK/Rem/ANI group; ^Ω^ *p* < 0.05 compared to MK/Rem/CXM group; **p < 0.01 compared to MK/Rem/DG group; ^+^ *p* < 0.05, ^++^ *p* < 0.01 compared to MK/Rem group.

In order to exclude a possibility that the amnestic effect of the inhibitors was produced by their influence on memory reactivation process rather than on reconsolidation, we administered the inhibitors immediately after the reminder. As depicted in Figure 2, administering ANI, CXM, or 2-D-Gal just after the reminder significantly decreased avoidance during the test indicating amnesia (Figure 2, Reminder/ANI (N = 23, *p* < .001 compared to the Control), Reminder/CXM (N = 20, *p* < .001 compared to the Control), and Reminder/DG (N = 22, *p* < .001 compared to the Control). However, prior administration of MK-801 prevented this amnestic effect (Figure 2, MK/Reminder/ANI (N = 15, *p* < .01 compared to Rem/ANI group), MK/Reminder/CXM (N = 15, *p* < .05 compared to the Rem/CXM), and MK/Reminder/DG (N = 16, *p* < .01 compared to Rem/DG groups).

## Discussion

The primary objective of our study was to explore the effects of an NMDA receptor antagonist in the model of reminder-associated amnesia in young chicks. Our findings demonstrate that memory impairment induced by either pre- or post-reactivation intracerebral administration of protein synthesis inhibitors (PSIs) such as anisomycin or cycloheximide, or a glycosylation inhibitor 2-deoxygalactose, can be effectively counteracted by prior intraperitoneal administration of the NMDA receptor antagonist MK-801.

In general, protein synthesis inhibitors such as anisomycin or cycloheximide have a greater effect on memory than NMDA receptor blockers such as MK-801. Thus, administration of MK-801 in the passive avoidance learning task in chicks at a dose of 0.2 mg/kg suppressed memory retention to 40% of avoidance (Burchuladze and Rose, 1992; Tiunova et al., 2020), while administration of anisomycin or cycloheximide suppressed it to 20% (Tiunova et al., 2020). This difference may depend in part on the relative doses of the substances administered and the corresponding degree of suppression of protein synthesis and NMDA receptor activity. But another explanation for this difference may also be that protein synthesis inhibitors have a much more general effect on the molecular memory consolidation cascade than NMDA receptor antagonists, which leave intact a number of other important synaptic mechanisms for memory consolidation (Rose, 2000; Gibbs and Summers, 2002; Hale and Crowe, 2002).

As protein synthesis inhibitors both anisomycin and cycloheximide inhibit translational process and were shown to effectively suppress it in chick’s brain (Bull et al., 1976). However, based on the effects of intracerebral and systemic injections, anisomycin was less effective in both protein synthesis suppression and in producing amnesia than cycloheximide (Bull et al., 1976). In mice, the level of protein synthesis suppression by anisomycin was only slightly lower than that of cycloheximide but the behavioral effect of ANI was weaker than that of cycloheximide (Flood et al., 1973).

The passive avoidance learning model in chicks has been extensively employed in studies of memory reconsolidation (Anokhin et al., 2002; Summers et al., 2003; Salinska, 2006; Crowe et al., 2008; Samartgis et al., 2012; Krolik et al., 2020). Reconsolidation of the reactivated memory was demonstrated to depend on protein and glycoprotein synthesis (Anokhin et al., 2002), RNA synthesis (Sherry et al., 2010), and both NMDA and non-NMDA glutamate receptors (Summers et al., 1997; Sherry and Crowe, 2008). The onset of amnesia, triggered by a reminder combined with various inhibitors, ranges from 30 min (CDK-5 inhibitor) to 90 min (non-NMDA receptors antagonist) post-reminder. In all cases studied, the recall deficit was transient, with spontaneous memory recovery occurring between 24 and 48 h post-reminder treatment (Anokhin et al., 2002; Summers et al., 2003; Crowe et al., 2008). In our current research, the reminder was administered 2 h post-training, with the retention test occurring 2 h post-reminder, i.e., 4 h after training. Previous studies have shown that at this time point, the recall level in chicks receiving reminder/anisomycin or reminder/2-deoxygalactose treatment was significantly lower than that in untreated and control groups (Anokhin et al., 2002). Importantly, administration of either anisomycin, cycloheximide, 2-deoxygalactose or MK-801 2 hours after the passive avoidance training without memory reactivation did not affect memory (Litvin and Anokhin, 2000; Anokhin et al., 2002).

Earlier, we also found that in the passive avoidance model, the retention deficit produced by memory reactivation coupled with an amnestic agent was transient. The deficit was observable up to 5 h after the reminder treatment but faded away by 24 h post-reminder (Anokhin et al., 2002). Thus, by 24 h after the start of reminder-associated amnesia memory in the “amnestic” groups (i.e., ANI/Rem, DG/Rem, CXM/Rem) would spontaneously recover and the avoidance levels would be approximately equal in all the groups. For this reason, we tested the chicks 2 h after the reactivation and did not examine 24-h memory retention in chicks with memory loss prevented (i.e., MK/ANI/Rem, MK/DG/Rem, MK/CXM/Rem) because even if extinction of the rescue effect had occurred, it would be masked by the spontaneous memory recovery.

In the present study we demonstrate that the reminder-associated retention deficit can be diminished by an NMDA receptor antagonist, MK-801.

The prevention of reminder-associated amnesia by NMDA receptor antagonists has been previously documented in various animal models, including rats in fear conditioning and snails in conditioned food aversion paradigms (Ben Mamou et al., 2006; Nikitin and Solntzeva, 2012). These studies show that pre-memory reactivation administration of NMDA receptor antagonists effectively averts the amnestic effects of post-reminder PSIs. In rats, pre-reminder infusion of an NMDA receptor antagonist ifenprodil alone had no effect on memory reconsolidation (Milton et al., 2013). However, interestingly, administration of NMDA receptor antagonists in snails resulted in amnesia, indicating a dependency of memory reconsolidation on both protein synthesis and NMDA receptor activity (Nikitin and Solntzeva, 2012). In our chick model, we observed similar paradoxical result: pre-reminder administration of MK-801 alone substantially diminished recall, yet its combination with pre- or post-reminder PSI resulted in a recall comparable to that of animals with unimpaired memory. This phenomenon, wherein the combined application of two amnestic treatments abolishes the effects of both, is particularly noteworthy.

NMDA receptors, and, in particular, their GluN2A and GluN2B subunits, were demonstrated to play a key role in the processes associated with memory reconsolidation. Recent studies have addressed the possible roles of individual NMDAR subunits during memory reactivation. These studies, examining the effects of NMDAR subunit antagonists in tasks such as object recognition and inhibitory avoidance extinction in rats (Radiske et al., 2021; Rossato et al., 2023), have revealed distinct roles for GluN2B and GluN2A subunits. Reactivation of conditioned fear memory in rats elevated GluN2B expression in the basolateral amygdala (Espejo et al., 2016). Moreover, administration of a GluN2B subunit antagonist ifenprodil into the basolateral amygdala prevented destabilization of conditioned fear memory in rats thus producing rescuing effect on the reactivated memory (Milton et al., 2013). The same effect was observed if ifenprodil was injected into thalamic nucleus reuniens that participates in the interaction of the hippocampus and cortical areas involved in conditioned fear memory (Alfei et al., 2021). On the other hand, antagonists of the GluN2A subunit did not affect destabilization processes but prevented subsequent restabilization of memory (Milton et al., 2013).

It has been suggested that memory destabilization (labilization) involves GluN2B-containing NMDA receptors, while subsequent stabilization (reconsolidation) requires GluN2A-containing receptors. This distinction may explain the varying effects of pre- and post-reactivation administration of non-selective NMDAR antagonist AP5, which appears to interfere with two distinct NMDAR-related processes: administration prior to reactivation prevents memory destabilization, whereas post-reactivation administration inhibits the reconsolidation of destabilized memory (Radiske et al., 2021; Rossato et al., 2023).

In contrast, in our study, pre-reactivation administration of the non-selective NMDAR antagonist MK-801 impaired memory recall. This discrepancy could be attributed to several factors, such as differences in learning models, the distinct nature of the NMDAR antagonist used (non-competitive in our case), or the method of antagonist administration (systemic *versus* local). Nonetheless, our findings underscore the efficacy of MK-801 in preventing amnesia induced by memory reactivation coupled with PSIs.

Two interpretative models emerge from these data. The first is based on the two-phase model of reconsolidation, suggesting that memory reactivation triggers an initial destabilization phase followed by a restabilization phase (Junjiao et al., 2019). According to this model, PSIs administered during memory reactivation prevent its restabilization, leading to the deterioration of the original engram. Pre-reminder administration of NMDA receptor antagonist MK-801 may have no effect on engram reactivation and behavioral expression of memory during the reminder session, but it could inhibit the destabilization of engram rendering the subsequent restabilization phase unnecessary (Ben Mamou et al., 2006). This can preserve the original engram despite administration of protein synthesis inhibitors.

An alternative explanatory approach, proposed by Nikitin and colleagues (Nikitin et al., 2020), views development of amnesia as an active process requiring specific mechanisms. This view states that protein synthesis is essential for establishing stable amnesia. Thus, administration of a PSI would inhibit the development of amnesia triggered by an NMDA antagonist during memory reactivation, thereby preserving the original memory. This hypothesis raises the difficult questions about how the brain differentiates between protein synthesis necessary for memory reconsolidation and that required for stabilizing amnesia. It is plausible that the mode of PSI action might depend on the brain state altered by MK-801 administration. On the other hand, the destabilization/restabilization model does not satisfyingly explain the amnestic effect of MK-801 alone if the NMDA receptor antagonist prevents only memory destabilization. We might therefore hypothesize a dual action for MK-801 in memory reconsolidation process: one that prevents protein synthesis-independent engram destabilization upon its retrieval and another that interferes with protein synthesis-mediated engram stabilization after its updating. Further investigations are needed to clarify the cellular and molecular mechanisms of this memory destabilization/restabilization process.

## Data Availability

The raw data supporting the conclusions of this article will be made available by the authors, without undue reservation.
